# Evaluating the Effectiveness of a Codeveloped e-Mental Health Intervention for University Students: Protocol for a Randomized Controlled Trial

**DOI:** 10.2196/49364

**Published:** 2023-08-30

**Authors:** Angel Y Wang, Melissa Vereschagin, Chris G Richardson, Hui Xie, Kristen L Hudec, Richard J Munthali, Lonna Munro, Calista Leung, Ronald C Kessler, Daniel V Vigo

**Affiliations:** 1 Department of Psychiatry Faculty of Medicine University of British Columbia Vancouver, BC Canada; 2 School of Population and Public Health Faculty of Medicine University of British Columbia Vancouver, BC Canada; 3 Faculty of Health Sciences Simon Fraser University Burnaby, BC Canada; 4 Department of Health Care Policy Harvard Medical School Boston, MA United States

**Keywords:** mental health, substance use, college students, digital interventions, randomized controlled trial, mobile phone

## Abstract

**Background:**

University life typically occurs during a period of life transition, where the incidence of mental health and substance use problems and disorders peaks. However, relatively few students obtain effective treatment and support. e-Interventions have proven effective in improving the psychological outcomes of university students and have the potential to provide scalable services that can easily integrate into existing models of care. *Minder* is a mobile app codeveloped with university students that offers users a collection of evidence-based interventions tailored to help university students maintain their mental health and well-being and manage their substance use.

**Objective:**

This paper describes the protocol for a randomized controlled trial (RCT) that aims to assess the effectiveness of the *Minder* app in improving the mental health and substance use outcomes of university students.

**Methods:**

This study is a 2-arm, parallel assignment, single-blinded, 30-day RCT with 1 intervention group and 1 waitlist control group. Overall, 1496 (748 per trial arm) university students from the University of British Columbia Vancouver Campus (N=54,000) who are aged ≥17 years, have a smartphone with Wi-Fi or cellular data, and speak English will be recruited via a variety of web-based and offline strategies. Participants will be randomized into the intervention or control group after completing a baseline survey. Those randomized into the intervention group will gain immediate access to the *Minder* app and will be assessed at 2 weeks and 30 days. Those randomized into the control group will be given access to the app content after their follow-up assessment at 30 days. The primary outcomes are measured from baseline to follow-up at 30 days and include changes in general anxiety symptomology, depressive symptomology, and alcohol consumption risk measured by the General Anxiety Disorder 7-Item scale, Patient Health Questionnaire 9-Item scale, and US Alcohol Use Disorders Identification Test-Consumption Scale, respectively. Secondary outcomes include measures related to changes in the frequency of substance use, mental well-being, self-efficacy in managing mental health and substance use, readiness to change, and self-reported use of mental health services and supports (including referral) from baseline to follow-up at 30 days.

**Results:**

Trial recruitment and data collection began in September 2022, and the completion of data collection for the trial is anticipated by June 2023. As of May 10, 2023, a total of 1425 participants have been enrolled.

**Conclusions:**

The RCT described in this protocol paper will assess whether the *Minder* app is effective in improving the mental health and substance use outcomes of a general population of Canadian university students. Additional secondary outcome research aims to explore additional outcomes of interest for further research and better understand how to support students’ general mental well-being.

**Trial Registration:**

ClinicalTrials.gov NCT05606601; https://clinicaltrials.gov/ct2/show/NCT05606601

**International Registered Report Identifier (IRRID):**

DERR1-10.2196/49364

## Introduction

### Background

University represents a period of transition where many students move away from home for the first time, requiring them to navigate the newfound independence and overcome unanticipated challenges when trying to make new friends and preserve existing relationships [1-3]. Students may find themselves suddenly dealing with new stressors without their previously relied upon support systems [4]. These stressors related to university life are combined with a period of development where the onset of many mental health and substance use disorders peaks [5,6]. Certain individuals may also be more likely to experience mental health problems in university based on various factors such as age, sexual orientation, ethnicity, and adverse childhood experiences [7]. Overall, this results in a population with a relatively high prevalence of mental health and substance use disorders [8,9]. For example, a study of college students across multiple countries found the 12-month prevalence of major depressive disorder, generalized anxiety disorder, and alcohol use disorder to be 18.5%, 16.7%, and 6.3%, respectively [9]. Despite the high prevalence of emerging mental health and substance use concerns, many university students do not seek help for these issues [10,11].

e-Interventions that promote self-management and link students to in-person services represent a promising means of addressing needs related to substance use and mental health issues. The potential of digital mental health interventions (eg, web-based programs and mobile apps) has been widely promoted, as they are often easily scalable and as beneficial as in-person therapies in promoting university students’ mental health [12-14]. Considering the high co-occurrence between mental health and substance use concerns [15], e-interventions are well positioned to offer low-barrier access to content supporting transdiagnostic approaches to interventions that acknowledge not only each individual concern but also their intersectionality. Existing digital interventions also address a range of challenges, including depression and anxiety [16], substance use [17], safety and risk management [18,19], and levels of severity (eg, subclinical mild to moderate symptom management and treatment for clinical conditions), and can be easily integrated into existing systems of care as a form of low-intensity, self-directed support [20].

### Objective

To address these needs, a mental health and substance use mobile app called *Minder* was codeveloped with and for Canadian university students. This codevelopment process involved significant input from students through the creation of a Student Advisory Committee (SAC), usability testing via a virtual bootcamp (ie, individual user testing combined with a web-based survey), focus groups, and a pilot feasibility study [14]. By combining the prevention and early intervention components, the *Minder* app has been designed to target the general population of university students. This paper describes the protocol for a pragmatic 2-arm randomized controlled trial (RCT) to assess the effectiveness of the *Minder* mobile app in improving university students’ mental health and substance use outcomes.

## Methods

### Study Objectives and Hypotheses

Our primary objective is to test whether the *Minder* intervention improves mental health and substance use outcomes in a general population of university students. Our null hypothesis is that there is no difference between the intervention group, who are given access to a full version of the *Minder* mobile app, and the control group, who are given access to a restricted version of the app that sends reminder notifications about baseline and follow-up surveys but prevents access to all intervention components. We hypothesize that assignment to the intervention group will result in greater effects on the primary mental health and substance use outcomes. As such, we expect that participants assigned to the intervention group will be more likely to experience improvements in their mental health and substance use outcomes than those in the control condition.

### Study Design and Setting

**Figure 1:**
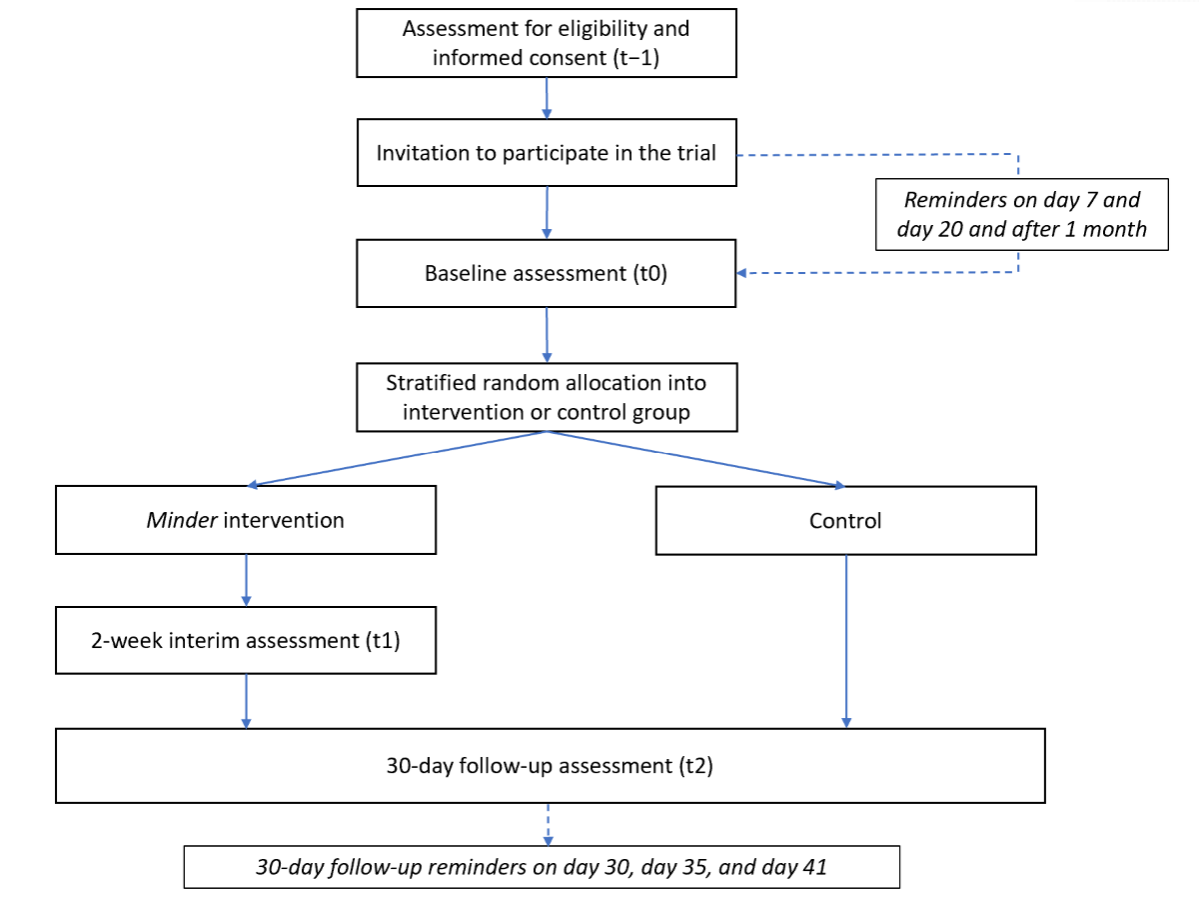
Outline of the trial process, including eligibility assessment, consent, baseline, randomization, and follow-up.

The study is a single-blinded, 2-arm parallel assignment RCT with 1 intervention group and 1 control group (Figure 1). In the pretrial period (t−1), all students are screened for eligibility and invited to enroll in the study; enrolled participants then complete the baseline assessment survey (t0). After randomization, all participants enter into the trial (with full access or restricted access to the *Minder* app, depending on group assignment), concluding with a follow-up assessment survey at 30 days (t2; see Table 1 for participant flow through the study). In addition to the baseline and follow-up surveys, the intervention group will also complete an assessment at 2 weeks. The baseline and follow-up assessments contain all primary and secondary outcomes, whereas the 2-week survey contains only 2 primary outcome measures (Patient Health Questionnaire 9-Item [PHQ-9] and General Anxiety Disorder 7-Item [GAD-7]). All assessments are delivered through the app and typically require approximately 15 minutes to complete but may be completed at the participant’s own pace. The 30-day follow-up assessment may be completed for up to 14 days after the 30-day study period end point.

Upon the completion of the randomized controlled trial, participants in the control group will be offered access to the intervention for ethical reasons and will be followed (with those already in the intervention group) for up to 6 additional months as part of a separate period of natural observation not included in the formal RCT analyses. This trial was registered with ClinicalTrials.gov (NCT05606601) on November 4, 2022. The study will be conducted at the University of British Columbia (UBC), Vancouver Campus. All data will be collected in Canada.

**Table 1:** Participant flow through the trial.

			Enrollment (t−1)		Study period				
					Baseline assessment and allocation (t0)		Interim assessment (t1; 2 weeks)		Follow-up assessment (t2; 30 days)
**Enrollment**									
	Eligibility screen	✓							
	Informed consent	✓							
**Group allocation and assessment**									
	*Minder* intervention			✓		✓		✓	
	Control			✓				✓	
**Assessment measures**									
	Anxiety symptoms (GAD-7^a^)			✓		✓		✓	
	Depressive symptoms (PHQ-9^b^)			✓		✓		✓	
	Alcohol consumption risk (USAUDIT-C^c^)			✓				✓	
	Opioid use frequency			✓^d^				✓	
	Stimulant use frequency			✓^d^				✓	
	Cannabis use frequency			✓				✓	
	Alcohol binge frequency (question 3, USAUDIT-C)			✓				✓	
	Alcohol use frequency (questions 1 and 2, USAUDIT-C)			✓				✓	
	Mental well-being (SWEMWS^e^)			✓				✓	
	Readiness to change			✓				✓	
	Self-efficacy: mental health			✓				✓	
	Self-efficacy: substance use			✓				✓	
	Connection to services and supports			✓				✓	

^a^GAD-7: General Anxiety Disorder 7-Item.

^b^PHQ-9: Patient Health Questionnaire 9-Item.

^c^USAUDIT-C: US Alcohol Use Disorders Identification Test-Consumption Scale.

^d^Baseline measure used for study group allocation.

^e^SWEMWS: Short Warwick-Edinburgh Mental Wellbeing Scale.

### Eligibility Criteria and Assessment

Participant eligibility will be determined through an eligibility screener delivered on Qualtrics, a web-based survey platform, before registering for the study.

Participants must meet all the following inclusion criteria: student currently enrolled at the UBC Vancouver campus, aged ≥17 years, have access to and be able to use a smartphone with Wi-Fi or cellular data, and speak English. Students will be excluded based on an eligibility screening item assessing suicidality risk (“We want to make sure that this app is appropriate for you at this time. Do you have a current suicidal plan [i.e., a plan to end your life]?”). Anyone endorsing a current suicidal plan (ie, answering “yes”) will not proceed with the registration form and will instead be provided with a list of crisis resources.

### Intervention

*Minder* was codeveloped with input from 225 university students, including 44 student project team members. Codevelopment occurred through engaging students and volunteers on the project team, a SAC, and quantitative and qualitative studies (web-based survey, pilot feasibility RCT, and focus groups). Feedback was used to guide the app revisions following each of these processes, resulting in a final app that differed considerably from the initial prototype. Details of each component of these adaptation processes can be found in the study by Vereschagin et al [14].

**Figure 2:**
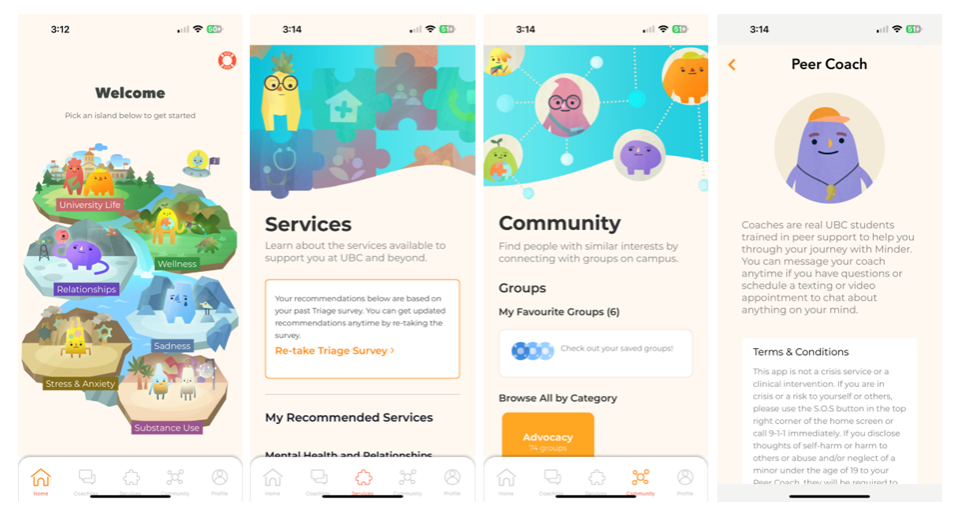
Minder main app components. The Minder app home page has 6 islands (University life, Relationships, Stress and Anxiety, Wellness, Sadness, and Substance Use) that deliver a variety of activities through an interactive chatbot. The Services component includes a short survey that assesses needs and preferences and matches users with resources. The Community component provides users with a directory of student groups and clubs they can filter through by interest. The Peer Coaching component matches users with a trained peer that provides psychosocial and app support.

*Minder* delivers a suite of evidence-based interventions that have been tailored to help university students improve their well-being and manage their substance use through 4 main components: Chatbot Activities, Services, Community, and Peer Coaching (Figure 2). The Chatbot Activities consist of an automated preprogrammed chatbot text and videos that draw primarily from cognitive behavioral therapy (CBT), given its demonstrated transdiagnostic efficacy in web-based intervention programs [21-24]. The Chatbot Activities also incorporate evidence-based content derived from metacognitive therapy, dialectical behavioral therapy, and motivational interviewing. These activities have been developed through collaboration with mental health professionals and university students so that the information can be presented in a way that is consistent with evidence-based practices and appropriate for this demographic. The Chatbot Activities were designed to support students on a range of topics and include content to manage mild to moderate distress and emotions and to help navigate general student life (eg, relationships, time management, and sleep hygiene). In addition, the content of the Chatbot Activities relates to the use of alcohol, cannabis, opioids, and stimulants, including general psychoeducation and motivational interviewing. These topics encapsulate many general skills included in CBT for managing emotions (eg, recognizing automatic thoughts) as well as additional topics highlighted by students through our codevelopment process as being more relevant to student well-being, such as transitioning to university. To increase the accessibility of the app, all activities that include video content have been closed-captioned in both English and French. Users have access to this entire range of content in the app and can choose to engage with any activities, regardless of their current mental health or substance use status. The Services component of *Minder* includes a validated psychosocial screening tool that provides users with tailored recommendations and resources based on their current needs and preferences [25]. It consists of a short survey with several categories (mental health, safety, substance use, abuse, housing, education, and sexual wellness) and an individually tailored list of relevant services that can be filtered by factors such as cost, availability, and delivery method. The Community component of *Minder* contains a searchable inventory of student groups or clubs at the university, which can be filtered according to the student’s interest. Finally, to support the use of the app, Peer Coaches (university student volunteers that have been trained by the research team’s clinicians) are available to help users navigate through different app components and provide nonclinical peer support. Once a Peer Coach is assigned, users can communicate with their coach through secure in-app texting or video calls in a synchronous or asynchronous manner. In this study, users in the intervention group will be offered Peer Coaches who will reach out to their assigned users twice during the study period (ie, at the beginning of the trial upon assignment and around the midpoint of the trial). Additional information on the development of *Minder* and its components can be found in the study by Vereschagin et al [14].

### Outcomes

#### Primary Outcomes

The primary outcomes are changes in general anxiety symptomology, depressive symptomology, and alcohol consumption risk from baseline to follow-up at 30 days.

##### Anxiety Symptoms

The GAD-7 scale is a commonly used self-report scale to assess symptoms of generalized anxiety. Questions on the scale draw from the Diagnostic and Statistical Manual of Mental Disorders, 4th edition [26], criteria for anxiety disorders and were formally validated against diagnostic psychiatric interviews [27]. Each question is scored from 0 (not at all) to 3 (nearly every day), with total scores ranging from 0 to 21 and higher scores indicating a worse outcome (ie, greater frequency of anxiety symptoms). In this study, changes in anxiety symptomology from baseline to follow-up at 30 days will be based on the GAD-7 total scores in both the intervention and control groups.

##### Depression Symptoms

The PHQ-9 scale is a self-report scale used to assess symptoms of depression and has been validated against diagnostic interviews administered by mental health professionals [28]. The 9 questions are based on the Diagnostic and Statistical Manual of Mental Disorders, 4th edition [26], diagnostic criteria for depressive disorders and scored on a scale of 0 (not at all) to 3 (nearly every day). The total scores range from 0 to 29, with higher scores indicating a worse outcome (ie, greater frequency of depression symptoms). In this study, changes in depressive symptomology from baseline to follow-up at 30 days will be based on the PHQ-9 total scores in both the intervention and control groups.

##### Alcohol Consumption Risk

The US Alcohol Use Disorders Identification Test-Consumption Scale (USAUDIT-C) is a 3-item self-report scale adapted from the consumption questions in the widely used Alcohol Use Disorders Identification Test (AUDIT). Compared with the AUDIT, the USAUDIT-C includes expanded response options for the first 3 AUDIT questions—from 5 to 7 categories—to allow for more precise measurements in accounting for differences in standard drink sizes and cut-off limits [29]. Although the USAUDIT-C was primarily adapted for US contexts, we believe the expanded response options will also allow for a more precise assessment of adherence to guidelines in drinking frequency (question 1), drinking quantity (question 2), and binge drinking (question 3) within Canadian contexts. For questions 1 and 3, scoring ranges from 0 to 6, where 0 represents “Never” and 6 represents “Daily” frequency. For question 2, scoring ranges from 0 to 6, where 0 represents “1 drink” and 6 represents “10 or more drinks” on a typical day when drinking. The total scores summed from all 3 questions range from 0 to 18, with higher scores indicating a worse outcome (ie, higher-risk level of excessive or harmful alcohol consumption). In this study, changes in alcohol consumption risk from baseline to follow-up at 30 days will be based on the USAUDIT-C total scores in both the intervention and control groups.

#### Secondary Outcomes

##### Overview

To provide further information on the underlying mechanisms and areas in which *Minder* is affecting participants’ overall well-being, a variety of secondary outcomes will be examined. Secondary outcomes include measures related to changes from baseline to follow-up at 30 days in the frequency of substance use (cannabis consumption, binge drinking, alcohol use, opioid use, and nonmedical stimulant use), mental well-being, self-efficacy managing mental health and substance use, readiness to change, and self-reported use (including referral) of mental health services and supports. An example of the questions used to assess secondary outcomes is provided in Multimedia Appendix 1.

##### Substance Use: Opioid Use Frequency

Changes will be based on the self-reported frequency of *both medical and nonmedical* opioid use in both the intervention and control groups.

##### Substance Use: Stimulant Use Frequency

Changes from baseline to follow-up at 30 days will be based on the self-reported frequency of nonmedical stimulant use in both the intervention and control groups.

##### Substance Use: Cannabis Use Frequency

Changes from baseline to follow-up at 30 days will be based on the self-reported frequency of cannabis consumption in both the intervention and control groups.

##### Substance Use: Alcohol Binge Frequency

Changes from baseline to follow-up at 30 days will be based on the responses to question 3 of the USAUDIT-C in both the intervention and control groups.

##### Substance Use: Alcohol Use Frequency

Changes from baseline to follow-up at 30 days will be based on the responses to questions 1 and 2 of the USAUDIT-C in both the intervention and control groups.

##### Mental Well-Being

Changes from baseline to follow-up at 30 days will be based on the total scores from the Short Warwick-Edinburgh Mental Wellbeing Scale in both the intervention and control groups. The Short Warwick-Edinburgh Mental Wellbeing Scale is a 7-item scale that has been widely validated, has strict unidimensionality, and is free from item bias [30]. The total scores range from 7 to 35, with higher scores indicating better outcome (ie, higher positive mental well-being).

##### Readiness to Change

Changes from baseline to follow-up at 30 days for the use of individual substances (alcohol, cannabis, opioids, and stimulants) will be based on self-reported readiness to change ladder assessments in both the intervention and control groups. The ladder assessments consist of a single item for each substance that asks participants to rate their readiness to change their use on a Likert-type response scale ranging from 0 (no thought of changing) to 10 (taking action to change). The response range parallels stages in the behavior change process from precontemplative to action stages, with higher scores indicating higher readiness to change and, therefore, a higher likelihood of changing their current substance use behavior. These questions were adapted from a readiness to change measure used by Merrill et al [31] to assess anticipated changes in alcohol use (adapted from The Contemplation Ladder, originally validated by Biener and Abrams [32]).

##### Self-Efficacy

Changes in mental health self-efficacy from baseline to 30-day follow-up are based on 5 survey questions in the intervention and control groups. Each question is scored separately using a Likert-type response ranging from 1 (not at all confident or likely) to 5 (totally confident or likely), with higher scores indicating a better outcome (ie, greater self-efficacy). Similarly, in both the intervention and control groups, changes in substance use self-efficacy from baseline to follow-up at 30 days will be based on 4 self-report survey questions assessing different aspects of self-efficacy related to substance use management. Each question is scored separately using a response ranging from 1 (not at all confident) to 5 (totally confident), with higher scores indicating a better outcome (ie, greater self-efficacy). Both the mental health and substance use self-efficacy survey questions were developed by the research team based on the theory of self-efficacy by Bandura [33].

##### Connection to Services and Supports

Changes in the use of mental health services and supports in both groups will be assessed by comparing responses to 2 yes-or-no survey questions from baseline to 30-day follow-up. The questions ask if participants have been diagnosed or treated by a professional for a list of mental health conditions and whether they have used any mental health treatments (medication, psychotherapy, mindfulness, and web-based tools) in the past 30 days or currently. Two additional survey questions are only asked at the 30-day follow-up: accessing types of support services and joining any university clubs or participating in any club-related events.

### Supplementary Feedback on User Experience

In addition to questions on the primary and secondary outcomes associated with the randomized controlled trial, participants in the intervention group will be asked a set of 12 additional questions on their experience using the *Minder* app at the end of the 30-day follow-up assessment. These questions will be used to inform the ongoing development of the app and use a combination of Likert-type and open text box responses to collect information on experiences using specific app components, reasons for using (or not using) features such as the Peer Coach, and any suggestions for improving key features such as the *Minder* chatbot.

### Sample Size and Power

The sample size calculation for a small effect assessed by the PHQ-9, GAD-7, and USAUDIT-C was calculated using an effect size defined by Cohen *d*=Δ/σ, where Δ is the group mean difference at the completion of the study and σ is the (pooled) within-group SD. For a small effect size (Cohen *d*=0.2), the sample size required to have 80% power at the level of significance *P*=.02 (0.05/3 primary outcomes) is 524 in each group. Assuming a 30% attrition rate, we require 748 participants in each group. To account for a conservative estimate of a small effect size and a 30% attrition rate, we estimate that a minimum of 748 randomized participants will be needed for each study group, totaling at least 1496 participants.

### Sample Recruitment

Sample recruitment will use a widespread, multifaceted approach that includes web-based and offline interactions with students as well as university faculty and administrators.

Web-based recruitment will occur through social media engagement via Facebook, Instagram, Twitter, and Reddit research laboratory or project pages or through recruitment material reposted by organizations represented on the SAC or UBC-affiliated groups (eg, student clubs and student government representatives). Web-based recruitment will also occur through the linked Student e-Mental Health survey, a version of the World Mental Health International College Student survey adapted for Canadian contexts and administered at UBC (approved by the UBC Behavioral Research Ethics Board under ethics ID #H19-02538 [34]). At the end of the linked survey, prospective participants will receive a message offering an opportunity to test a mental health and substance use app, and interested students will be redirected to a registration form for this study where they can consent and register to participate if qualified.

Offline recruitment will include in-person booths of student team members in frequented communal areas with high student traffic (eg, student union and main campus lawn or pathway) around the UBC Vancouver campus; placing recruitment posters in public places on campus; and distributing mini posters with QR codes to classes, students, and student groups on campus.

Student engagement will also occur through collaboration with select UBC faculty members and administrators. Specifically, members of the research team will attend in-person and web-based courses at the university to provide students with a short presentation about the study and will request that recruitment materials be included on course web pages and emails. In addition, student groups at the university will be contacted to share recruitment information with their members through emails and newsletters or to coordinate with study team members attending group meetings or events to directly share information about the study. Invitations to participate in this study will also be sent to students who have previously expressed interest in participating in research studies and asked to be contacted if study opportunities with the research laboratory became available.

Widespread signage will also be implemented through posters at on-campus bus stops and digital screens in on-campus buildings, as well as printed posters within buses designated for routes with direct connections to the university.

### Randomization and Study Arms

A study participant is defined as an individual who has completed the baseline survey and has been randomly assigned into either the control or intervention group. Participants are randomized using a stratified block randomization function built into the app. The randomization list for the built-in function was created using the web-based stratified block randomization list creator in the clinical trial software Sealed Envelope (Clerkenwell Workshop) [35]. To ensure that each arm of the randomized trial contains an equal number of participants endorsing a history of substance use, participants will be assigned to either group A (substance use: any medical opioid use, nonmedical opioid use, or nonmedical stimulant use) or group B (nonsubstance use), based on their answers to the opioid and stimulant use questions in the baseline survey. After being stratified into group A or group B, individuals are then automatically randomly assigned (via the randomization list embedded into the app) to either the control or intervention arm of the trial using a 1:1 ratio in blocks of 10.

Those randomized into the control group will receive a message saying that they will be asked to complete an additional survey at 30 days and will only receive full access to the app after that. During the 30-day study period, the control group participants will receive a restricted version of the app that only permits access to the following: an introduction video with several students providing a broad overview of the different aspects of the app and its potential value, several onboarding slides with a short summary of the main app components, and baseline and follow-up assessment surveys plus reminder notifications to complete those surveys. Participants randomized into the intervention group will receive immediate access to the full *Minder* app.

### Strategies to Improve Adherence to Intervention Protocols

All participants will receive both email reminders and in-app push notifications to complete the assessment surveys. More specifically, after the initial email to prospective participants with instructions to download the app and complete the baseline survey (to be randomized), an email reminder to complete the baseline survey will be sent on day 7 and again on day 20 to those who have not yet completed it. Participants who have not completed the baseline after 1 month may receive an additional email reminder. Participants assigned to the intervention group will then receive an email and push notification to complete the 2-week survey. Participants in both the intervention and control groups will receive an email and push notification 30 days after baseline completion and a group assignment to notify them to complete the follow-up survey, along with a reminder email 5 days and 11 days later if the survey has still not been completed.

Participants in the intervention group will also receive general in-app push notifications on days 4, 18, and 24 to highlight the different features of the app. The intervention group also receives a push notification when they have a new in-app message from their assigned Peer Coach, either a scheduled message (at assignment and around the study midpoint) or a reply to an ongoing text conversation with the participant.

### Data Management and Withdrawal

All data are collected in the app and stored on secure servers located in Canada. The research team will download all study data regularly (business days excluding statutory holidays) and monitor for any issues with data collection. Access to the data will be limited to the members of the research team. To ensure that participant information and privacy are protected, the *Minder* app backend will abide by the UBC Research Ethics Board guidelines and British Columbia’s Freedom of Information and Protection of Privacy Act. Participant data are collected using a unique study ID value within the system to ensure that survey response data and app use data remain separate from identifying information contained in the participant registration and remuneration documentation. The only exception will be if a participant elects to withdraw from the study and indicates their decision to have their data deleted; in this case, the data manager will use the unique study ID value to link their registration information with their survey response data and app use data and delete all their data from the system and all saved files.

### Data Analysis Plan

#### Statistical Analysis

The primary analysis will be intention to treat (ITT) to estimate the causal effects of the assignment to treatment on the trial outcomes for all participants. A participant within the study is defined as an individual who has completed the baseline assessment survey and has been randomized into either the control or intervention group. The analysis will consider the following 2 features of the trial design: 3 primary end points and the method of randomization. The effects of some of these design features (ie, randomized blocks) are expected to be small and statistically nonsignificant. Nonetheless, we will use multilevel regression and SAS (version 9.4; SAS Institute Inc) and R (version 4.3.1; R Foundation for Statistical Computing) software in our analyses to control for these effects.

Before any testing, we will compare the baseline differences between the randomization groups on demographic (eg, sex, gender, and ethnicity), socioeconomic (eg, year of study), and personal risk variables (eg, mental health diagnoses and use of mental health treatments). Any baseline prognostic variable with a standardized difference between the groups of Cohen *d*>.05 will be considered a candidate variable to control for in the analysis. The selection of Cohen *d*>.05 is arbitrary: there is no accepted threshold to determine a meaningful difference between the 2 groups. However, this is a very small effect and indicates approximately a 4% nonoverlap between the 2 populations.

For the 3 primary end points (PHQ-9, GAD-7, and USAUDIT-C), a global test with the null hypothesis of no treatment difference in all primary end points between the control and treatment groups will be conducted using a marginal multivariate regression model for correlated data on PHQ-9, GAD-7, and USAUDIT-C. Compared with testing each outcome separately, the advantages of the global test from joint modeling include more parsimonious hypothesis tests and mitigated concern of multiple testing issues [36-38] as well as pooling of the information over the correlated outcomes to increase study power, especially with missing outcomes [39]. When the global test rejects the null hypothesis and we conclude that there is an intervention effect on at least 1 of the 3 end points, we will then analyze each end point separately to identify which of the 3 study end points are affected by the intervention. We will use the sequential Hochberg correction method [40] to control the overall family-wise error rate at α=.05 when testing the hypothesis for each individual primary end point.

The general approach for analyzing all the types of study outcomes separately (including the 3 primary end points and the secondary end points) will be the generalized linear mixed-effect models (GLMMs) for clustered measures, with the randomization block as the clustering variable. These models can handle a wide range of outcome types, including continuous, binary, ordinal, and count, and can account for the correlations among observations within the same cluster. Specifically, we will analyze each primary end point using a linear mixed-effect model, a special case of the GLMM. For secondary outcomes, we will use linear mixed-effect models to analyze mental well-being measures and readiness to change measures, mixed logit models to analyze the connection to services and support-related measures (binary: yes or no), mixed-effects quasi-Poisson regression models with the log link (a special case of GLMMs) for substance use frequency measures, and mixed-effects proportional odds ordinal logistic regression (another special case of GLMMs) for self-efficacy measures. The treatment effect on an outcome at 30 days will be assessed using a GLMM with the treatment allocation as the main explanatory variable and with adjustment for baseline outcome assessment value and other baseline prognostic factors as fixed effects and the randomization block as a random effect. By properly specifying the GLMM, a wide range of scientific hypotheses can be tested, including the primary ones regarding treatment effect estimation. The quality of statistical inferences will be substantiated through rigorous model checking and validation techniques. When statistical models assume that residuals at certain levels follow normal distributions, this assumption will be checked using a normal probability plot, in which the ranked residuals are plotted against corresponding points on a normal distribution curve. When substantial departures from the normal residual assumption are found, robust empirical SE estimates that are valid with nonnormal residuals will be used for statistical inference. We will use SAS software (version 9.4; SAS Institute Inc) and R (version 4.3.1; R Foundation for Statistical Computing) for the statistical analyses of our primary and secondary outcomes. Subgroup analysis will be performed using the GLMM on the subsets of data formed by the subgroup.

Missing data will take two forms: (1) missed questions or items and (2) unit nonresponse or attrition (withdrawals and lost to follow-up). We anticipate that most missing data are owing to attrition, which leads to unit nonresponse at the 30-day assessment. To reduce bias and avoid the loss of statistical power in our analysis of primary and secondary outcomes, we will use likelihood-based direct estimation, which is equivalent to performing multiple imputations under the model to predict values for missing data. One advantage of using the abovementioned multilevel GLMMs for analyzing primary and secondary outcomes is that it uses all the available data by including those study units with missing outcome values and it produces valid inference under the assumption of missing at random, which is a more relaxed assumption of missing data mechanism than missing completely at random. The missing values will be regarded as missing at random when the nonresponse behavior is believed to be a function of observed data, particularly common when assessing mental health and substance use behaviors and using a digital self-report format. The potential bias of these inferences will be quantified if these missing data were suspected to be nonrandom even after conditioning on the observed data, using the methods [41,42] developed by our team member and implemented in R package *isni* [43]. In summary, state-of-the-art statistical methods will be used to address these missing data issues.

In addition to the primary analysis using ITT, we will conduct a secondary analysis to evaluate the Complier Average Causal Effect (CACE) of the intervention. The primary analysis using ITT estimates the causal effects of treatment assignment on the trial outcomes for all participants, regardless of whether those assigned to the intervention actually use the *Minder* app (ie, complying with the treatment assignment). The results from the ITT analysis will be useful to policy makers and inform the effects of applying the treatment program to the population. Although ITT analysis is informative and valid, one limitation of the ITT analysis is that it estimates the program effectiveness rather than method efficacy. The program effectiveness depends on the proportion of compliers, which can change (eg, students change their willingness to use the intervention after they learn that the intervention works). Thus, to complement this approach, we will also conduct a CACE analysis that will focus on the intervention efficacy of those who actually use the e-intervention. The CACE analysis in conjunction with the abovementioned ITT analysis will provide a more complete picture of the intervention effect. By identifying who uses and benefits from the intervention, the trial can yield findings of direct interest to the scientific community and individual students. To overcome the challenge that the study participants’ compliance and app use behaviors are self-selected in nature and not randomized, we will use the principal stratification approach to estimate the CACE effects on multiple end points in RCTs with treatment noncompliance [36] and the dose-response relationship between the extent of app use (eg, completed Chatbot Activities and number of contacts with Peer Coach) and intervention effect [44]. Given the diverse nature of the target population combined with the self-guided use of intervention components, we also plan to examine heterogeneity in treatment effects using a series of exploratory analyses that use sociodemographic (eg, gender and year of study) and assessment data (eg, symptom level on PHQ-9 and GAD-7) collected at baseline to examine the impact of the *Minder* app on key subgroups.

#### Masking

The study will use a single-blinded approach where only the investigators (including the statistician) will be blinded to the treatment group assignment when examining the primary hypotheses.

### Continued Monitoring

There will be no stopping rules in this study—the anticipated benefits and unanticipated adverse effects are not life threatening and too small to merit such an approach. In addition, there will be no sequential analyses of the data: the analyses will commence at the end of the trial when the data manager provides the statistician with a blinded database for the analyses. Of note, data from the 2-week assessment completed by the intervention group will be removed from the data set used for the primary outcome analysis to maintain the blinding of the statistician.

### Ethics Approval

This study was approved by the UBC Behavioral Research Ethics Board on January 6, 2022 (ethics ID: H21-03248). Any changes to the protocol will be submitted for approval to the UBC Research Ethics Board.

### Consent

Consent will be obtained using a web-based UBC Qualtrics form before the email validation process (ensuring current student enrollment and only 1 study registration per student), which provides access to the app and baseline assessment survey.

## Results

The trial was registered on ClinicalTrials.gov (NCT05606601) on November 4, 2022. Recruitment and data collection began in September 2022. As of May 10, 2022, a total of 1425 participants have been enrolled. The results of the study will be available at the end of 2023 and will be published in international and national journals and presented at conferences.

## Discussion

### Expected Findings

Postsecondary students have been found to have high rates of mental health and substance use problems but many of these students do not access mental health or other professional supports [10]. e-Interventions have been shown to be effective among university students and may help address barriers to seeking support by providing tools that are easily accessible and can be scaled up for university campuses [16]. This manuscript describes the study protocol for a RCT to assess whether the *Minder* app is effective in improving mental health and substance use outcomes in a general population of Canadian university students. We hypothesize that individuals assigned to the intervention group will be more likely to experience improvements in mental health and substance use outcomes compared with individuals in the control condition. Additional secondary outcomes and analyses seek to inform potential mechanisms through which future studies can be conducted to better understand how to support the mental well-being of students and support the ongoing improvement of the *Minder* app as an effective digital mental health tool.

### Limitations

Although many similar e-interventions have experienced large loss to follow-up and poor participant engagement, the incorporation of Peer Coaches for each participant in addition to well-spaced reminders to return to the app and complete baseline and follow-up surveys are expected to improve app engagement and reduce loss to follow-up. It is also important to note that this intervention was designed for use in the general population with mild symptomology so recruitment may be challenging as these students may be less interested than a clinical population in participating in a mental health study. We have addressed this challenge by using a comprehensive set of recruitment methods to engage potential participants in the broader university population. The intervention also includes components relevant to all students, including content addressing general well-being and more clinically relevant content. Students who choose to participate in this study may also be more motivated than the general population to seek help or to improve their mental health or substance use concerns. This may impact their willingness to participate and responses to self-report surveys, which may lead to reporting bias; however, we believe that these biases will not differ across the surveys or between intervention and control groups. Moreover, mental health and substance use concerns are high among university populations, with a large fraction experiencing psychological distress; therefore, a study sample reporting a range—including elevated levels—of mental health or substance use concerns is representative of students’ functioning, which supports the generalizability of the results to the broader student population. In addition, participants in the intervention group will be unblinded, which could lead to biased self-reports of mental health symptomology related to placebo effects and social desirability. Although our use of standardized assessments completed via nonidentifiable web-based surveys should help mitigate some of these risks, it will be important to address this limitation in future studies on the *Minder* app by incorporating a sham or placebo control in future trials and examining the cognitive mechanisms associated with targeted outcomes.

### Conclusions

Despite these limitations, the findings from this trial will inform the ongoing refinement of the *Minder* app. One key aspect of the *Minder* app that separates it from other similar tools is its extensive codevelopment process with students, especially in the development of content that is presented in a chatbot manner that is more interactive than other modular, web-based CBT interventions. It is designed to support a general population of students across many potential topics, whereas many existing tools target specific issues (eg, depression or anxiety). Thus, by designing a tool that can be used by all students, we hope to provide a suite of interventions (eg, from time management to managing emotions) that builds resilience across a range of conditions and improves the well-being of students as they face the many challenges associated with postsecondary education and young adulthood. This includes the tailoring and ongoing improvement of content using focus groups and continued engagement with specific groups of university students who may have more unique university experiences, including international students; lesbian, gay, bisexual, transgender, queer, 2-spirit, intersex, asexual, and similar minority (LGBTQ2IA+) students; and Indigenous and other racialized students. Through further analyses of app use data and subgroup analyses, we hope to identify areas of particular interest and need to help us better understand how to improve and scale up the delivery of the app to other student populations.

## Appendix

Multimedia Appendix 1 Secondary outcome questions.

## Abbreviations

AUDIT: Alcohol Use Disorders Identification Test
CACE: Complier Average Causal Effect
CBT: cognitive behavioral therapy
GAD-7: General Anxiety Disorder 7-Item
GLMM: generalized linear mixed-effect model
ITT: intention to treat
LGBTQ2IA+: lesbian, gay, bisexual, transgender, queer, 2-spirit, intersex, asexual, and similar minority
PHQ-9: Patient Health Questionnaire 9-Item
RCT: randomized controlled trial
SAC: Student Advisory Committee
UBC: University of British Columbia
USAUDIT-C: US Alcohol Use Disorders Identification Test-Consumption Scale

## Data Availability

The data sets generated during this study are not publicly available in accordance with our ethics approval. Interested researchers are advised to contact the corresponding author for further information on gaining access to anonymous data as further approval by the relevant research ethics boards may be needed.
