# Supplementary material for: Evaluating the Effectiveness of a Codeveloped e-Mental Health Intervention for University Students: Protocol for a Randomized Controlled Trial
Source: JMIR Res Protoc. 2023 Aug 30;12:e49364. doi: 10.2196/49364 (PMC10500355; doi:10.2196/49364)
Supplement: Multimedia Appendix 1 [file resprot_v12i1e49364_app1.docx]

## Appendix A. Secondary Outcome Questions

*Substance Related Questions*

- In the past 30 days, how often did you use cannabis?
  - Not in the past 30 days
  - 1 day in the past 30 days
  - 2 or 3 days in the past 30 days
  - 1 or 2 day(s) per week
  - 3 or 4 days per week
  - 5 or 6 days per week
  - Every day: 1 or 2 times a day
  - Every day: 3 or more times a day
  - I don’t know
  - I prefer not to answer
- *Thinking back over the past 30 days:*
  - How often do you have a drink containing alcohol?
    - Never
    - Less than monthly
    - Monthly
    - Weekly
    - 2-3 times a week
    - 4-6 times a week
    - Daily
  - How many drinks containing alcohol do you have on a typical day when you are drinking?
    - 1 drink
    - 2 drinks
    - 3 drinks
    - 4 drinks
    - 5-6 drinks
    - 7-9 drinks
    - 10 or more drinks
- How often do you have 5 (if your sex assigned at birth was male) OR 4 (if your sex assigned at birth was female) or more drinks on one occasion?
  - - Never
    - Less than monthly
    - Monthly
    - Weekly
    - 2-3 times a week
    - 4-6 times a week
    - Daily
- Have you used any of the following opioids in the past 30 days? Please select all that apply:
  - Pharmaceutical opioid taken as prescribed by a healthcare professional
  - Pharmaceutical opioid bought over-the-counter and taken as recommended
  - Pharmaceutical opioid taken without a prescription or in larger doses than prescribed/recommended to get high, buzzed, numbed out, or for any other reason
  - Any street opioid
  - I have not used any of the above in the past 30 days

For each selected response:

- - - In the past 30 days, how often did you use any xyz (replace with selected response):
      - Never
      - 1-3 days a month
      - 1-2 days a week
      - 3-4 days a week
      - Every or nearly every day

- In the past 30 days, how often did you use any street stimulant:
  - Never
  - 1-3 days a month
  - 1-2 days a week
  - 3-4 days a week
  - Every or nearly every day

*Mental Wellbeing Questions*

- *Below are some statements about feelings and thoughts. Please tick the box that best describes your experience of each over the last 2 weeks.*
  - I’ve been feeling optimistic about the future
    - None of the time; Rarely; Some of the time; Often; All of the time
  - I’ve been feeling useful
    - None of the time; Rarely; Some of the time; Often; All of the time
  - I’ve been feeling relaxed
    - None of the time; Rarely; Some of the time; Often; All of the time
  - I’ve been dealing with problems well
    - None of the time; Rarely; Some of the time; Often; All of the time
  - I’ve been thinking clearly
    - None of the time; Rarely; Some of the time; Often; All of the time
  - I’ve been feeling close to other people
    - None of the time; Rarely; Some of the time; Often; All of the time
  - I’ve been able to make up my own mind about things
    - None of the time; Rarely; Some of the time; Often; All of the time

*Self-Efficacy Sample Questions*

- How confident are you that you could effectively manage any mental health problems you experience (e.g., stress, anxiety or depression)?
  - 1 (Not at all confident), 2, 3, 4, 5 (Totally confident)
- How confident are you that you could manage your mental health without reaching out to the health system or other support services?
  - 1 (Not at all confident), 2, 3, 4, 5 (Totally confident)
- How confident are you that you would know how to access mental health services if you felt you needed them?
  - 1 (Not at all confident), 2, 3, 4, 5 (Totally confident)
- How likely are you to reach out to mental health services if you felt you needed them?
  - 1 (Not at all confident), 2, 3, 4, 5 (Totally confident)
- How confident are you that you can manage your mental health problems without using alcohol or other drugs as a coping strategy?
  - 1 (Not at all confident), 2, 3, 4, 5 (Totally confident)

*Readiness to Change Sample Question*

- Each rung of this ladder represents where a person might be in thinking about changing their drinking. Select the number that best represents where you are now.


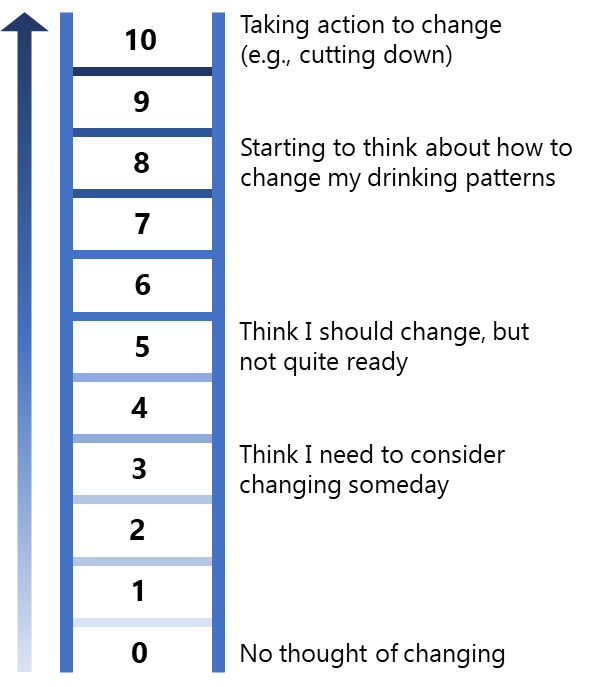


*Connection to Services and Support Questions*

- Have you ever been diagnosed or treated by a professional for any of the following? Check all that apply:
  - Depression
  - Anxiety, Panic, or Phobias
  - Post-Traumatic Stress Disorder
  - Obsessive-Compulsive Disorder (OCD)
  - Personality Disorders
  - Attention Deficit-Hyperactivity Disorder (ADD/ADHD)
  - Autism Spectrum Disorders
  - Bipolar Disorder
  - Schizophrenia/Psychosis
  - Eating Disorders
  - Alcohol Use or Abuse
  - Drug Use or Abuse
  - Other, please specify: [TEXT BOX]
  - I have never been diagnosed with any of the above-mentioned disorders
  - Prefer not to say

- Have you utilized any of the following mental health treatments in the past 30 days?
  - Medication prescribed by a health professional
    - Yes or No
  - Psychotherapy or counseling with a health professional (for example, CBT, DBT etc)
    - Yes or No
  - Mindfulness, meditation or other forms of self-guided therapies
    - Yes or No
  - Online tools or apps to support your mental health
    - Yes or No
  - Other [TEXT BOX]
- Have you accessed any of the following types of support services in the past 30 days? Check all that apply:
  - Abuse (e.g. including physical and sexual violence)
  - Sexual wellness
  - Education
  - Housing and Food Security
  - Other, please specify: [TEXT BOX]
  - Prefer not to say
- Have joined any university clubs or participated in any club-related events in the past 30 days?
  - Yes or No

*Feedback on User Experience Questions*

- Did you have access to the app and all of its features over the last month?
  - Yes or No
- Did you interact with your peer coach at all over the past month?
  - Yes or No

*IF NO:*

- Can you explain briefly why you didn’t utilize the peer coach?

[TEXT BOX]

*IF YES:*

*Please rate how much you agree with each of the following statements about your interactions with your* peer coach*.*

- My peer coach helped me navigate the app.
- Strongly Agree, Agree, Disagree, Strongly Disagree
- I used the app more because of my peer coach.
- Strongly Agree, Agree, Disagree, Strongly Disagree
- My peer coach sessions provided me with emotional support.
- Strongly Agree, Agree, Disagree, Strongly Disagree
- The peer coach support I received was an important part of my overall experience with the app.
- Strongly Agree, Agree, Disagree, Strongly Disagree
- Overall, did you find the Minder app easy to use?
  - 1 (Definitely not); 2; 3; 4; 5 (Definitely yes)
- Check below which of the following features were most helpful in your experience with the Minder app. Please select all that apply.
- Minder chatbot
- Services
- Community
- Peer Coach
- Reminders
- SOS Button

IF ANY RESPONSE IS SELECTED ABOVE:

- What did you like best about these features?

[TEXT BOX]

- Did you try any of the activities from the Substance Use map?
  - Yes or No

*IF YES:*

- What did you like most about these activities? What could be improved?

[TEXT BOX]

*IF* NO:

- Why not?
- I wasn’t sure what was included in this section
- I didn’t think the content would be helpful
- I didn’t think I needed these tools
- Other: [Free text]
- Did you try the Minder chatbot?
  - Yes or No

IF YES:

- How could we improve the Minder chatbot?

[TEXT BOX]

*Please indicate how much you agree with each of the following statements about the Minder app.*

- Using the Minder app helped me manage my mental health.
- Strong Agree, Agree, Disagree, Strongly Disagree
- Using the Minder app helped me manage my alcohol use.
- Strong Agree, Agree, Disagree, Strongly Disagree
- Using the Minder app helped me manage my drug use.
- Strong Agree, Agree, Disagree, Strongly Disagree
- Using the Minder app helped me feel more connected to others.
- Strong Agree, Agree, Disagree, Strongly Disagree
- Using the Minder app helped improve my overall well-being.
- Strong Agree, Agree, Disagree, Strongly Disagree
- I would recommend the Minder app to a friend.
- Strong Agree, Agree, Disagree, Strongly Disagree
